# Supplementary material for: Agreement of image quality metrics with radiological evaluation in the presence of motion artifacts
Source: MAGMA. 2025 Jun 10;38(6):991–1002. doi: 10.1007/s10334-025-01266-y (PMC12638400; doi:10.1007/s10334-025-01266-y)
Supplement: Supplementary file 1 — (pdf 1324 KB) [file 10334_2025_1266_MOESM1_ESM.pdf]

Agreement of Image Quality Metrics with Radiological Evaluation in the Presence of Motion Artifacts

Elisa Marchetto[1,2,3†], Hannah Eichhorn[4,5†], Daniel Gallichan[3], Julia A. Schnabel[4,5,6], Melanie Ganz[7,8*]

[1]Bernard and Irene Schwartz Center for Biomedical Imaging, Dept. of Radiology, NYU School of Medicine, NY, USA.
[2]Center for Advanced Imaging Innovation and Research (CAI^{2}R), Dept. of Radiology, NYU School of Medicine, NY, USA.
[3]CUBRIC, School of Engineering, Cardiff University, Cardiff, UK.
[4]Institute of Machine Learning in Biomedical Imaging, Helmholtz Munich, Neuherberg, Germany.
[5]School of Computation, Information and Technology, Technical University of Munich, Munich, Germany.
[6]School of Biomedical Engineering and Imaging Sciences, King's College London, London, UK.
[7]Department of Computer Science, University of Copenhagen, Copenhagen, Denmark.
[8]Neurobiology Research Unit, Copenhagen University Hospital, Copenhagen, Denmark.

*Corresponding author(s). E-mail(s): melanie.ganz@nru.dk;
†These authors contributed equally to this work.

Supplementary Information

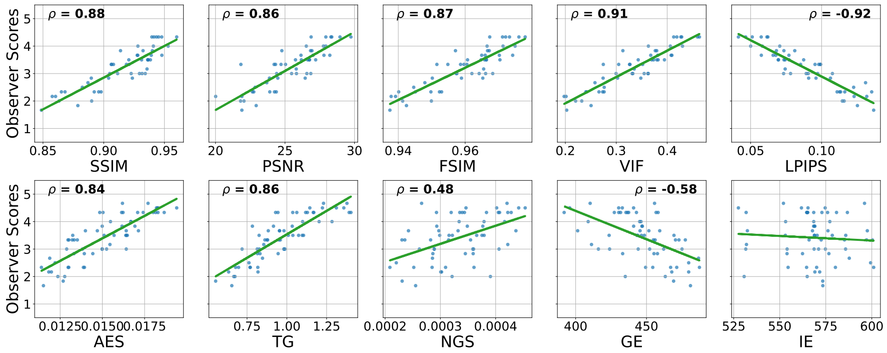

(a) T_2 FLAIR

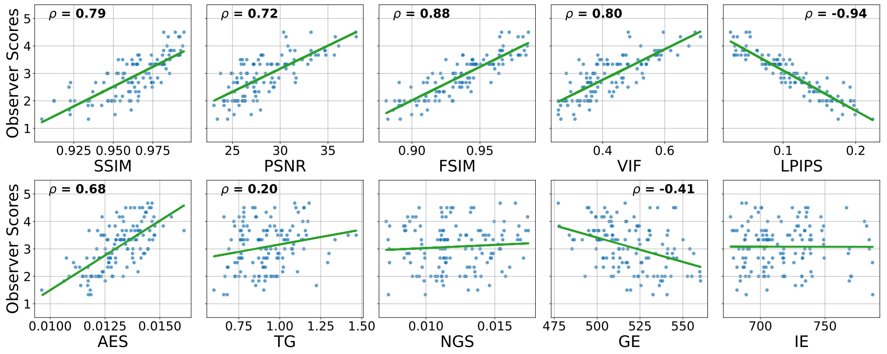

(b) T_1 TIRM

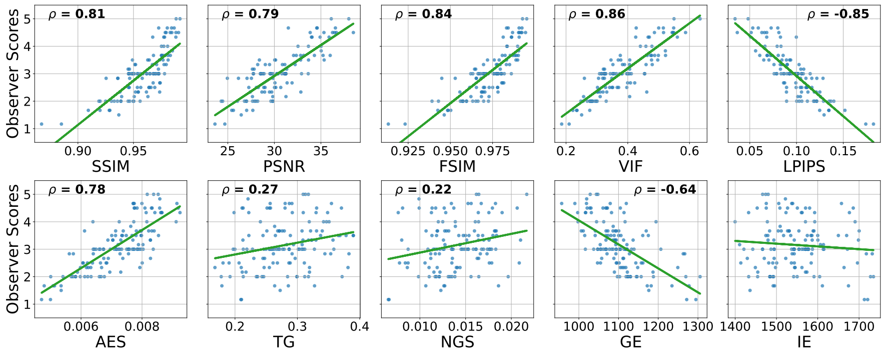

(c) T_2 TSE

Fig. S1: Scatter plots visualizing the distribution of metric values against observer scores for the T_2 FLAIR, T_1 TIRM, and T_2 TSE sequences of the NRU dataset. Each blue dot represents one image volume, and the corresponding regression line is shown in green. For statistically significant correlations (p-value < 0.05), the corresponding Spearman correlation coefficient is provided on top of each plot. The metrics were calculated with the pre-processing settings {*Multiply, Percentile, Worst*}. Non-integer observer scores result from averaging the scores across the four raters.

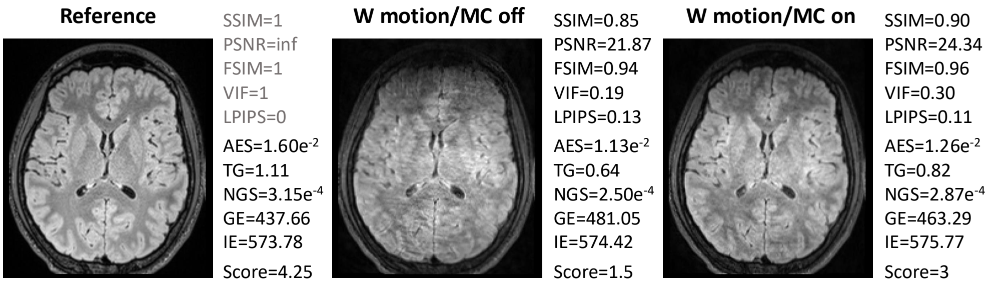

(a) T_2 FLAIR

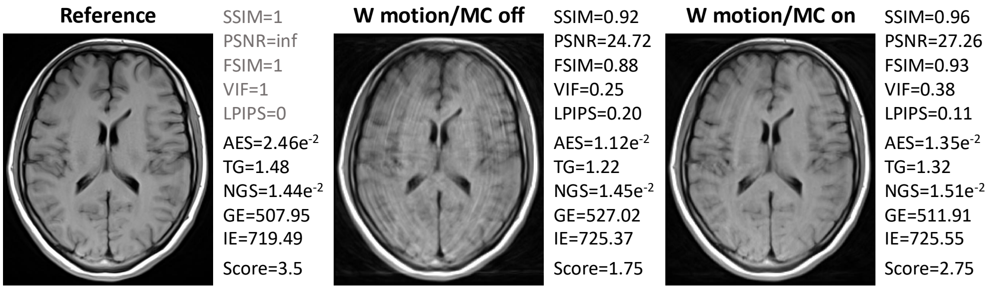

(b) T_1 TIRM

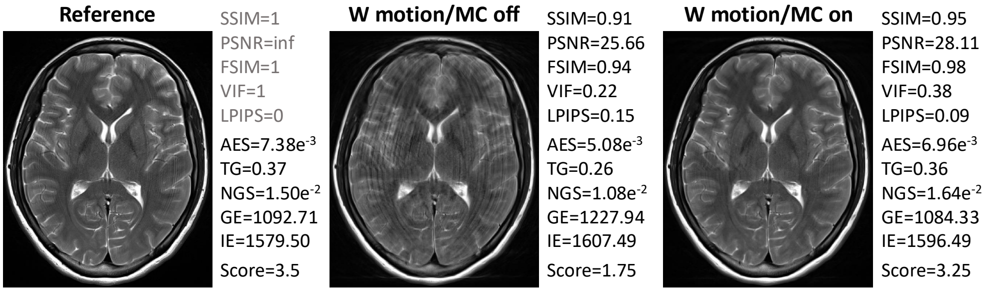

(c) T_2 TSE

Fig. S2: Examples images from one subject acquired using T_2 FLAIR (a), T_1 TIRM (b) and T_2 TSE (c). The reference image was acquired without voluntary motion and without motion correction, while the other two examples were acquired with voluntary motion (nodding/shaking) and with/without motion correction. Image quality metrics are reported, alongside with the average observers' evaluation scores ("Score"). The reference-based IQMs (SSIM, PSNR, FSIM, VIF, LPIPS) are colored in light-gray for the "Reference" image, followed by the reference-free metrics (AES, TG, NGS, GE, IE).

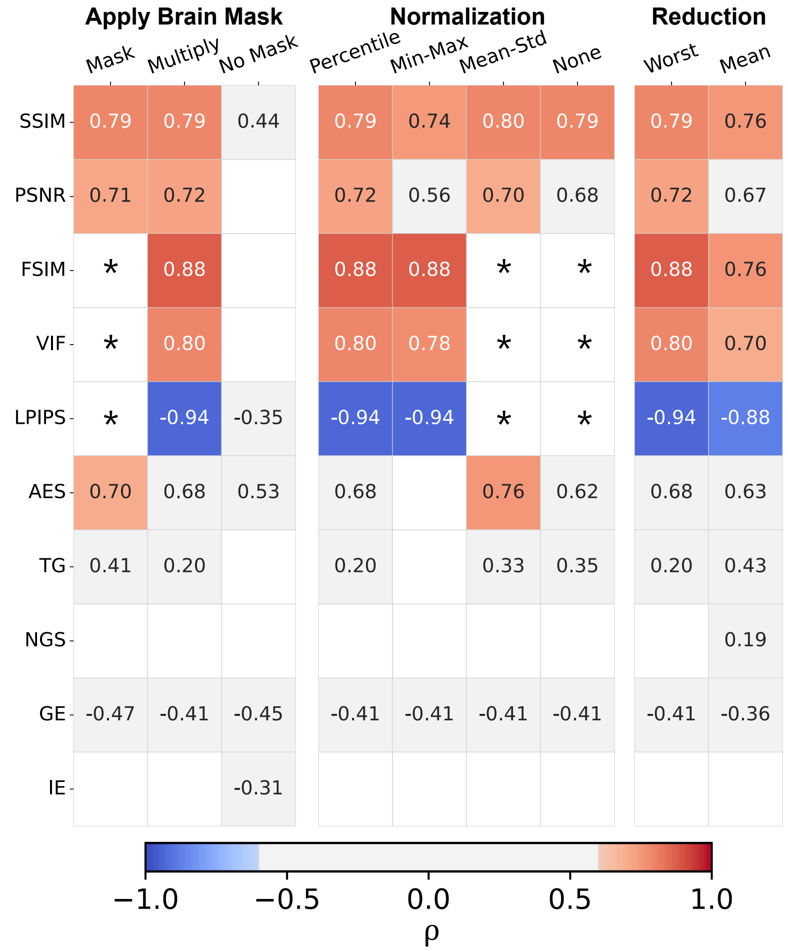

Fig. S3: Overview on the effect of pre-processing implementations in the correlation between IQM and observers' scores for the T_1 TIRM sequence from the NRU dataset. The table only shows statistically significant correlations ($p < 0.05$), leaving the box empty if this requirement is not fulfilled. We indicated with a "$*$" values for FSIM, VIF and LPIPS which are not available in case of normalization using "Mean-Std" and "None", as they require a specific range of values (see Table 1). Similarly, these values are unavailable with the "Mask" setting, as the metrics are computed across the entire matrix.

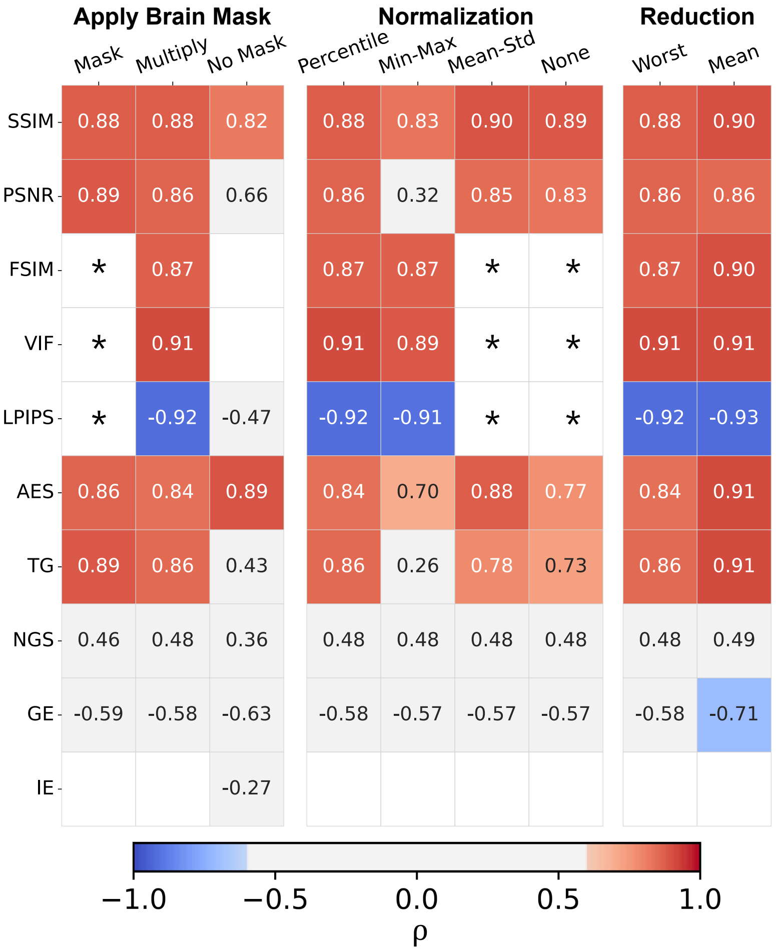

Fig. S4: Overview on the effect of pre-processing implementations in the correlation between IQM and observers' scores for the T_2 FLAIR sequence from the NRU dataset. The table only shows statistically significant correlations ($p < 0.05$), leaving the box empty if this requirement is not fulfilled. We indicated with a "$*$" values for FSIM, VIF and LPIPS which are not available in case of normalization using "Mean-Std" and "None", as they require a specific range of values (see Table 1). Similarly, these values are unavailable with the "Mask" setting, as the metrics are computed across the entire matrix.

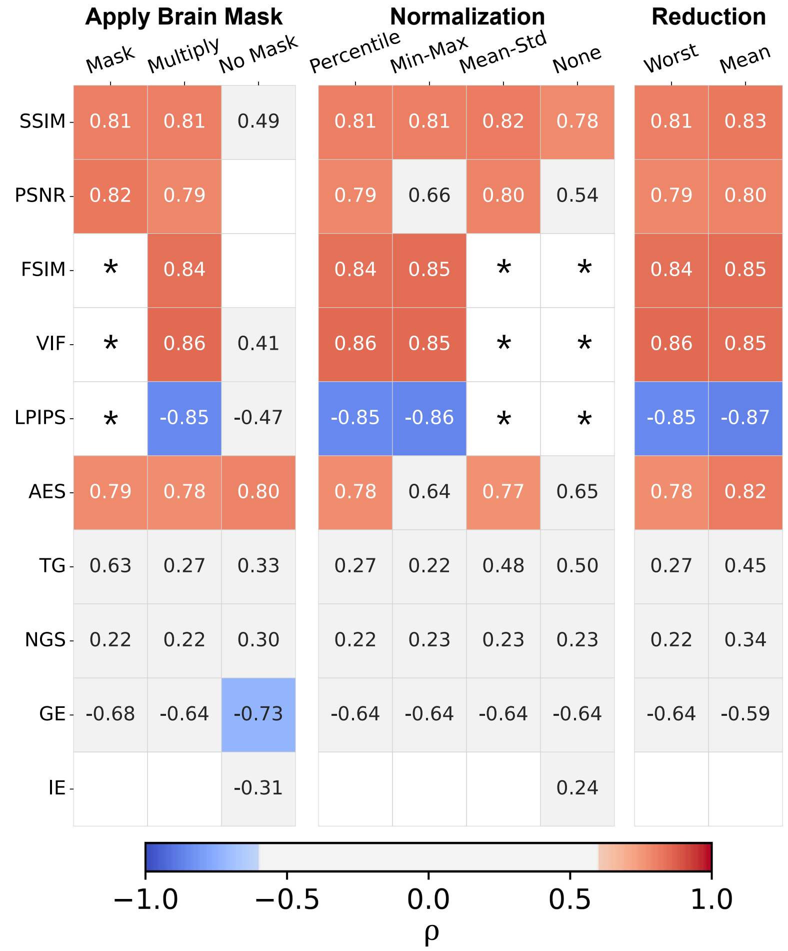

Fig. S5: Overview on the effect of pre-processing implementations in the correlation between IQM and observers' scores for the T_2 TSE sequence from the NRU dataset. The table only shows statistically significant correlations ($p < 0.05$), leaving the box empty if this requirement is not fulfilled. We indicated with a "$*$" values for FSIM, VIF and LPIPS which are not available in case of normalization using "Mean-Std" and "None", as they require a specific range of values (see Table 1). Similarly, these values are unavailable with the "Mask" setting, as the metrics are computed across the entire matrix.
